# Patterns of convergence in the central nucleus of the inferior colliculus of the Mongolian gerbil: organization of inputs from the superior olivary complex in the low frequency representation

**DOI:** 10.3389/fncir.2013.00029

**Published:** 2013-03-06

**Authors:** Nell B. Cant

**Affiliations:** Department of Neurobiology, Duke University Medical CenterDurham, NC, USA

**Keywords:** auditory pathways, binaural, hearing, neuroanatomy

## Abstract

Projections to the inferior colliculus (IC) from the lateral and medial superior olivary nuclei (LSO and MSO) were studied in the gerbil (*Meriones unguiculatus*) with neuroanatomical tract-tracing methods. The terminal fields of projecting axons were labeled via anterograde transport of biotinylated dextran amine (BDA) and were localized on series of horizontal sections through the IC. In addition, to make the results easier to visualize in three dimensions and to facilitate comparisons among cases, the data were also reconstructed into the transverse plane. The results show that the terminal fields from the low frequency parts of the LSO and MSO are concentrated in a dorsal, lateral, and rostral area that is referred to as the “pars lateralis” of the central nucleus by analogy with the cat. This region also receives substantial input from both the contralateral and ipsilateral cochlear nuclei (Cant and Benson, 2008) and presumably plays a major role in processing binaural, low frequency information. The basic pattern of organization in the gerbil IC is similar to that of other rodents, although the low frequency part of the central nucleus in gerbils appears to be relatively greater than in the rat, consistent with differences in the audiograms of the two species.

## Introduction

The inferior colliculus (IC) receives input from most of the auditory nuclei in the brainstem, as well as from a number of areas in the forebrain, including the auditory cortex (reviewed, e.g., in Casseday et al., 2002; Malmierca, 2005). In the cat, in which these projections have been studied in the most detail, it has been established that the terminal fields formed by the multiple inputs are not distributed homogeneously throughout the nucleus (e.g., Roth et al., 1978; Brunso-Bechtold et al., 1981; Kudo, 1981; Henkel and Spangler, 1983; Oliver, 1984, 1987; Shneiderman and Henkel, 1987; Shneiderman et al., 1988; Oliver et al., 1997; Loftus et al., 2004, 2010). The apparent partial or complete segregation of terminal fields formed by different sources of input supports the concept of synaptic domains in which specific neuronal populations in the IC form synaptic connections with only a subset of the total inputs to the IC (Oliver and Huerta, 1992; Oliver, 2000, 2005).

The purpose of the present study is to describe the distribution of terminal fields formed by inputs from the lateral and medial superior olivary nuclei (LSO and MSO) in the IC of the gerbil, a rodent commonly used in auditory research. Although the intrinsic organization of the IC of the rodent appears grossly similar to that in the cat, there are important differences (e.g., rat: Faye-Lund and Osen, 1985; Loftus et al., 2008). As rodents become more and more common in studies of the central auditory system, it is important to compare and contrast the details of the termination patterns in their IC with those established in the cat. Similar to results in the cat, differential termination of inputs from some of the major afferent sources to the IC have been reported in rodents (e.g., projections from the cochlear nucleus: Oliver et al., 1999; Malmierca et al., 2005; Cant and Benson, 2008; projections from the superior olivary complex: Fathke and Gabriele, 2009; Saldaña et al., 2009; projections from the nuclei of the lateral lemniscus: Gabriele et al., 2000; commissural projections: Malmierca et al., 2009; projections from the auditory cortex: Saldaña et al., 1996; Bajo and Moore, 2005), but there are no detailed published descriptions of the terminal distribution of the inputs from the LSO and MSO, two brainstem nuclei that extract binaural cues important for sound localization and other perceptual processes. In this paper, terminal fields formed in the gerbil IC by projections from the LSO and MSO are described and related to patterns of intrinsic organization. The results are consistent with the conclusion that the gerbil IC is organized according to the common plan proposed by Loftus et al. (2008). The extent to which the gerbil IC *appears* different from that of the cat or rat may be explained by a differential representation of specific frequency ranges in each species.

## Materials and methods

### Animals and tracer injections

#### Animals

This paper contains a description of the axonal termination patterns in the inferior colliculi of seven cases taken from a large collection with tracer injections in either the IC itself or in nuclei of the SOC. In all cases, female gerbils (*Meriones unguiculatus)* were obtained from Charles River Laboratories at approximately 8 weeks of age. They were housed in the Duke University Medical Center animal facilities until use. All procedures using these animals were approved by the Duke University Institutional Animal Care and Use Committee and were in accordance with NIH guidelines. The animals were deeply anesthetized for all surgical procedures and for the terminal perfusion.

#### Tracer injections in the IC or SOC and histological procedures

Three cases chosen from those described by Cant and Benson (2006) are used here to describe the intrinsic organization of the gerbil IC. The procedures for the surgery, injections, perfusions, and post-perfusion histological procedures were reported in detail in that paper. Very briefly, for the injections, gerbils were anesthetized with Nembutal (i.p., 50–70 mg/kg). A small hole was made in the skull and 10% biotinylated dextran amine (BDA) in 0.9% saline was injected iontophoretically through a glass pipette inserted into the IC. After survival periods of 5–11 days, the animals were given an overdose of Nembutal. When respiration ceased, they were perfused through the heart with a 4% paraformaldehyde fixative. Sections through the brain were cut at 40 μm and processed in 2 alternating series. One set of every other section was processed for visualization of BDA, and the second set was processed for cytochrome oxidase (CO) histochemistry. The procedures for injection of BDA into either the LSO or MSO in another four cases were exactly the same except for the location of the injection sites.

### Acquisition and manipulation of images

#### Digital photography

Digital images of all BDA- and CO-reacted sections were collected with a Zeiss Axiocam HRc camera attached to a Zeiss Axioscope 2 and controlled by Zeiss Axiovision software. The BDA sections were magnified through a 10× Plan-Apochromat objective, passed through a camera adapter with a magnification factor of 0.63×, and photographed at a resolution setting of 2600 × 2060 pixels (high resolution). The CO sections were magnified through either a 2.5× Plan-Neofluar or a 5× Plan-Apochromat objective, passed through the same adapter, and photographed at a resolution setting of 1300 × 1030 pixels (low resolution). All subsequent processing of these images was done in Photoshop CS4 running on Apple Macintosh computers.

#### Procedures for relating the sections from the experimental brains to a standard atlas

In a previous report (Cant and Benson, 2005), we described an atlas of the gerbil IC in which we established a coordinate system relating sections in the horizontal, transverse, and sagittal planes, referred to here as “the atlas.” In the present study, the atlas coordinates were transferred to the experimental cases through a series of systematic steps. First, images of the sections reacted for CO histochemistry were paired with sections from comparable levels of the atlas and were oriented and resized to give the best possible fit. The IC is a surface structure, and distortions of its superficial conformation often occur during histological procedures. Internal structural relationships appear to be much less affected by distortion. Therefore, landmarks such as differences in levels of CO activity within the IC, the border formed by the fibers of the commissure of the IC, the obvious boundary of the periaqueductal gray layer, the orientation of the midline, and the caudal boundaries of layers of the superior colliculus carried more weight in the matching process than did the exact contour of the surface. The procedure was constrained in two ways: (1) The spacing of sections was maintained in the experimental series. That is, once level H120 (for example) was established, levels H40 and H200 had to be represented by the CO sections immediately ventral and dorsal to it, respectively. (2) Once the best percent change in size was established for a given case, all sections had to be resized by the same amount, and resizing was always uniform. Through this procedure, the atlas coordinates for the appropriate plane were transferred to each CO-reacted section.

In a second series of steps, each experimental BDA-reacted section was re-sized and oriented to match its adjacent CO-reacted section (by convention, for a horizontal series, the CO section ventral to it and for a transverse series, the CO section rostral to it). Again, resizing was uniform, and the same percent change was used for every section in a given case. After the CO and BDA sections were matched, the coordinate grid of the atlas could be superimposed onto the BDA sections. (A brief summary of the procedure is illustrated in Cant and Benson, 2008). The final step was to group the BDA-reacted sections into a stack in which they were automatically lined up based on the atlas coordinates that had been applied to each one. These image stacks were used for the procedures described below.

#### Reconstructions from the horizontal to the transverse plane based on “Photoshop drawings”

Six of the seven cases described here were cut in the horizontal plane, which is a relatively unfamiliar plane for most readers. Both to make the results easier to visualize in three dimensions and also to facilitate comparisons across cases, the horizontally sectioned colliculi were reconstructed into the more commonly portrayed transverse plane. Although the reconstructions could be accomplished using the original digital images, the results are easier to compare and represent on the printed page if the original images are converted to black and white. Photoshop offers a way to do this that results in an image that superficially resembles a drawing made at the microscope but that is fast enough to make it practical to “draw” all of the sections through each IC. To make the drawings, the original color digital images were converted to grayscale and the stamp filter (under the sketch filter menu) was applied to each image. (Smoothness was always set to 1, but the light/dark balance was adjusted from case to case depending on the exposure and background staining in that case.) The stamp filter finds edges in the image and strokes them (O'Quinn, 2001), producing a black and white image that, especially at low magnification, looks similar to a drawing made by hand. (Note that this procedure works well only on very high resolution images; this is the reason that the digital images of the BDA sections were made at a resolution higher than that needed for routine visualization.) To complete the drawing, the surface contour of each section was traced by hand (i.e., by mouse) and applied to the filtered image. The images obtained in this way will be referred to as “Photoshop drawings.”

To make the reconstructions from the horizontal plane to the transverse plane, a “slice” 80 μm thick (one interval on the atlas grid) was selected at a given transverse level on each horizontal image in the image stack. The set of slices was copied to a file containing the chosen transverse atlas section, and each slice was positioned at the appropriate horizontal level. This procedure was repeated for each transverse level in the atlas (see Results). (Although not illustrated here, sections cut in the transverse plane could be reconstructed into the horizontal plane using the same procedure.) Because all of the cases are referenced to the same atlas coordinates, comparisons among cases are facilitated.

## Results

### Note on nomenclature

In order to avoid excessive and potentially confusing use of terminology based on relative position (i.e., lateral, rostral, etc.), I have employed nomenclature used in descriptions of the IC of other species but not applied previously to the gerbil. First, I will refer to a portion of the dorsolateral and rostral central nucleus as the *pars lateralis*. This designation is adopted from the classic description of the cat IC (Morest and Oliver, 1984; Oliver and Morest, 1984). (This region probably also corresponds to the part of the rat IC referred to as the “lemniscal zone” by Faye-Lund and Osen, 1985). Second, nomenclature introduced for the rat and guinea pig (e.g., Saldaña and Merchán, 1992, 2005; Malmierca et al., 1995) will be employed to refer to the axonal plexuses labeled when BDA is injected into the IC itself.

### Patterns formed by commissural connections in the gerbil IC

**Figure 1:**
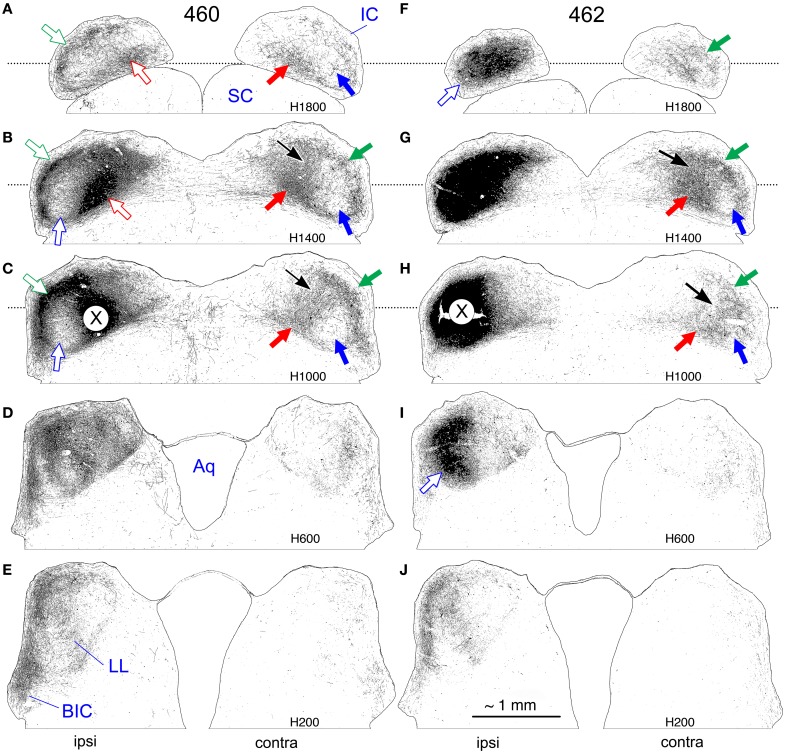
**Photoshop drawings of five evenly-spaced horizontal sections from cases 460 (A–E) and 462 (F–J).** The more dorsal sections are located at the top **(A,F)** and the more ventral sections are located at the bottom **(E,J)**. The caudal aspect of the IC is oriented toward the top. The corresponding atlas levels (H1800-H200) are indicated on each section. The dotted lines behind the top three rows indicate the approximate location of transverse level 960 (T960) of the IC atlas. The approximate locations of the injection sites in each case are indicated by the large X **(C,H)** on the right IC (located more precisely in Cant and Benson, 2006). Abbreviations: Aq, cerebral aqueduct; BIC, brachium of the inferior colliculus; contra, contralateral; IC, inferior colliculus; ipsi, ipsilateral; LL, lateral lemniscus; SC, superior colliculus. Arrows are referenced in the text. Scale bar (panel **J**) indicates approximately 1 mm and applies to all panels.

**Figure 2:**
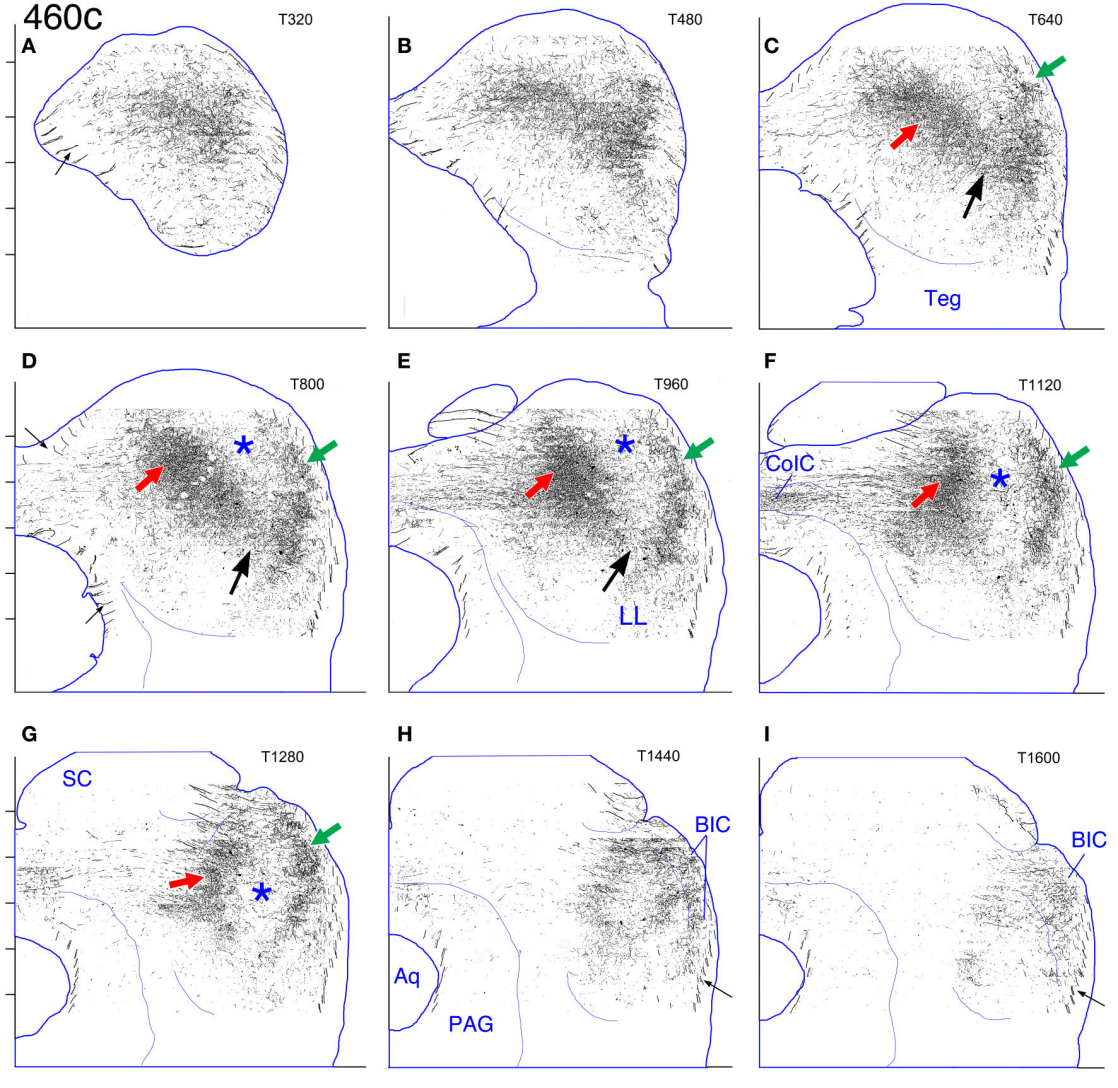
**Case 460c. (A–I)** Reconstruction of the contralateral (left) IC after a BDA injection in the right IC. In this and also in Figures 3, 6, and 9–14, nine evenly-spaced reconstructions of the IC are located on the corresponding atlas sections from caudal (panel **A**) to rostral (panel **I**). The dorsal direction is toward the top of each panel; the lateral direction is to the right; the midline is indicated by the line at the left of each panel. The transverse levels (T320, T480, etc.) indicated in the upper right of each panel refer to the atlas (see text). The outline of the *atlas section* at each level is drawn in blue. The short horizontal marks to the left of the ordinate for panels **(A,D**, and **G)** are 400 μm apart and indicate the levels of the horizontal sections shown in Figure 1 (i.e., from dorsal to ventral, H1800 to H200). For reasons of clarity, given the low magnification of the figures, all filled pixels *outside* the blue outlines were erased. (A little information in the reconstructions is lost because of this procedure since the contour lines [e.g., **(A,D,I)**, small arrows] for some of the stacked layers lie outside the blue outlines.) Regardless of whether the reconstructed IC was from the left or right side of the brain, all cases were plotted on atlas sections representing the right IC. (For those cases in which the left IC is reconstructed, as in this figure, the images were reflected about the midline.) Abbreviations: Aq, cerebral aqueduct; BIC, brachium of the inferior colliculus; CoIC, commissure of the inferior colliculus; PAG, periaqueductal gray matter; SC, superior colliculus; Teg, subcollicular tegmentum. Large arrows and asterisks are referenced in the text.

**Figure 3:**
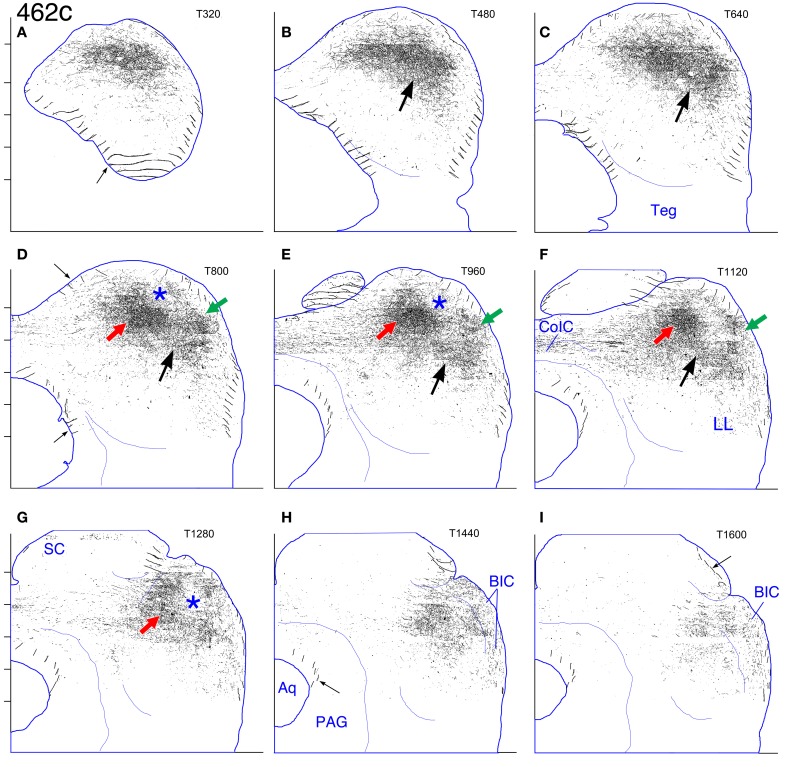
**Case 462c. (A–I)** Reconstruction of the contralateral (left) IC after BDA injection in the right IC. Details as for Figure 2.

**Figure 4:**
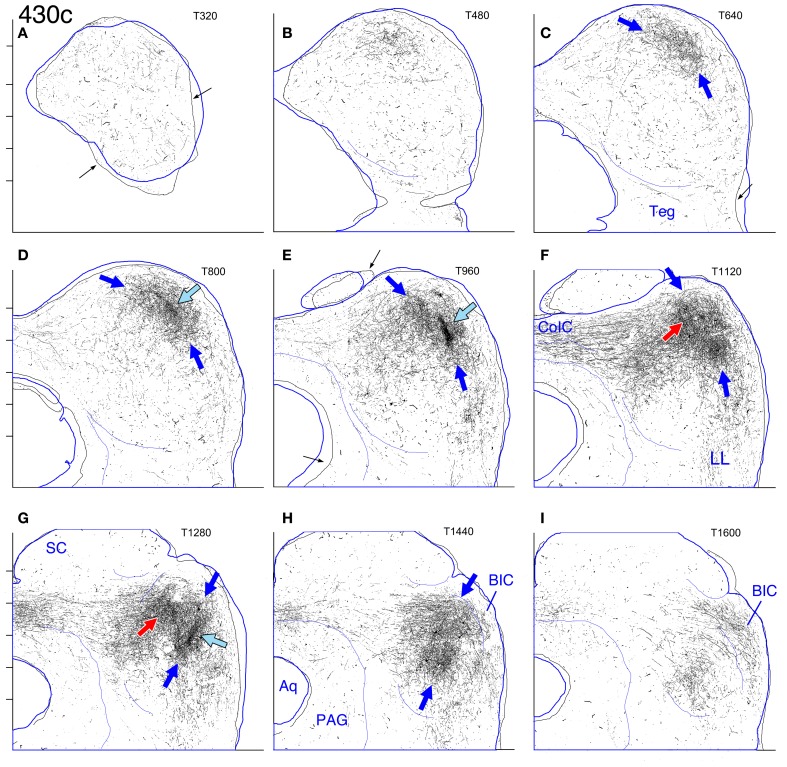
**Case 430c. (A–I)** Photoshop drawings of transverse sections through the contralateral (left) IC after a BDA injection in the right IC. Nine evenly-spaced sections were matched to transverse atlas sections. As in the other figures, the outlines of the atlas sections are shown in blue; the outlines of the sections from case 430 are indicated by the black outlines (e.g., small black arrows on panels **A,C**, and **E**). Other details as for Figure 2.

To provide a context for the description of the results of tracer injections in the SOC, the labeling patterns seen after tracer injections in the IC itself are presented for three cases (Figures 1–4). As in the rat (Saldaña and Merchán, 1992, 2005) and guinea pig (Malmierca et al., 1995), injection of an anterograde tracer into one IC gives rise to a characteristic pattern of labeling that reveals the topographic organization on both sides of the auditory midbrain.

#### Case 460 (Figures 1A–E, 2)

The center of the BDA injection site in this case (Figure 1C) was judged to be located in the “middle frequency” portion of the IC on the basis of the location of the labeled cells in the contralateral cochlear nucleus (Cant and Benson, 2006). In both inferior colliculi, labeled axon terminals are densely concentrated in two plexuses as described in other species. In the contralateral IC (where the pattern is not partially obscured by the injection site itself), the external (or lateral) plexus (Figures 1B,C, green arrows), lies just beneath the lateral surface and part of the caudal surface. The main (or medial) plexus (Figures 1A–C, red arrows) is most dense rostrally but extends caudally, where it appears to meet the caudal extension of the lateral plexus (Figures 1B,C, thin black arrows). The sparsely labeled rostrolateral area that lies between the two plexuses in this case is the pars lateralis of the central nucleus (Figures 1A–C, blue arrows). Where visible, the same pattern is evident on the ipsilateral side, with an external plexus (Figures 1A–C, open green arrows) and a main plexus (Figures 1A,B, open red arrows). As on the contralateral side, the ipsilateral pars lateralis (Figures 1B,C, open blue arrows) is relatively sparsely labeled even though it is quite close to the center of the injection site.

Reconstructions of the IC of case 460 into the transverse plane (Figure 2) make it easier to appreciate that the pattern in the gerbil is similar to that in other rodents. At middle levels through the IC, the main plexus (e.g., Figures 2C–E, red arrows) lies at an angle of approximately 45 degrees (with respect to the horizontal) in a location compatible with a presumed frequency representation in the middle range; rostrally, the main plexus assumes a more vertical orientation (Figures 2F,G, red arrows). The external plexus (Figures 2C–G, green arrows) lies lateral and caudal to the central nucleus of the IC and follows the curve of its external surface. In sections from about 25 to 50% of the caudal-to-rostral extent of the IC, a connection between the main and external plexuses is evident ventrally (Figures 2C–E, black arrows), but in more rostral sections (Figures 2F,G), the plexuses do not appear connected. In the most caudal sections, the two plexuses also appear connected (Figures 1A–B), but, in these sections, it is difficult to distinguish the rostral plexus from the caudal extension of the lateral plexus in the transverse plane. The dorsolateral and rostral part of the IC lying between the two main plexuses is relatively unlabeled in this case (Figures 2D–G, blue asterisks); it is this part of the IC that is referred to here as the pars lateralis.

#### Case 462 (Figures 1F–J, 3)

The injection site in this case was located at approximately the same dorsal-to-ventral and rostral-to-caudal level as that in 460 but was situated slightly more laterally (compare Figure 1H to Figure 1C). Based on the distribution of labeled cells in the cochlear nucleus, the injection site was judged to be centered in the low frequency representation of the IC (Cant and Benson, 2006).

In the contralateral IC, the external plexus (Figures 1F–H, green arrows) and main plexus (Figures 1G,H, red arrows) are both shifted in position compared to those in case 460 (dorsally and dorsolaterally, respectively). An apparent connection between the two plexuses (Figures 1G,H, thin black arrows) is shifted laterally and rostrally and lies in the pars lateralis of the central nucleus (Figure 1H, blue arrow). The pattern on the ipsilateral side is hidden by the injection site except in the most dorsal and ventral sections, where it can be seen that the pars lateralis is filled with labeled axons and terminals (Figures 1F,I, open blue arrows).

In the reconstruction of case 462 (Figure 3), the external (Figures 3D–F, green arrows) and main (Figures 3D–G, red arrows) plexuses on the contralateral side appear less obviously separate compared to case 460 (Figure 2); they are located more dorsally and are closer together. A connection between them lies in the pars lateralis (Figures 3C–F, black arrows), and, as for case 460, two distinct plexuses are difficult to distinguish in more caudal sections. Rostrally, the main plexus, while oriented vertically, does not extend quite so far ventrally as in case 460 (compare Figure 3G to Figure 2G).

#### Case 430 (Figure 4)

The center of the injection site in this case was located on a medial-to-lateral and a dorsal-to-ventral line with that of case 462 (Figure 1H) but was located rostral to it (illustrated in Cant and Benson, 2006). Based on the location of labeled cells in the cochlear nucleus (as well as in the MSO and LSO), the injection was centered in the part of the IC that represents the lowest frequencies processed in the gerbil IC. In contrast to the pattern in cases 460 and 462, two laminar plexuses are not clearly distinguishable in the contralateral IC. Rather, throughout most of the IC, an elongated plexus occupies pars lateralis, extending along part of its dorsal to ventral extent (Figures 4C–H, dark blue arrows). At rostral levels, the plexus reaches almost to the ventral boundary of the central nucleus (Figures 4G,H, dark blue arrows). Although a distinct lateral plexus cannot be identified at any level, a main plexus appears in the rostral IC (Figures 4F,G, red arrows), occupying a location comparable to that occupied by the main plexus in case 462 (compare Figure 4F to Figure 3F, red arrows). The density of terminal labeling within the plexus of labeled axons in pars lateralis is not uniform. Particularly obvious is a dense accumulation of terminals at some levels that appears to almost bisect the elongated axonal plexus (Figures 4D,E,G, light blue arrows).

### Projections into the IC from the lateral and medial superior olivary nuclei

**Figure 5:**
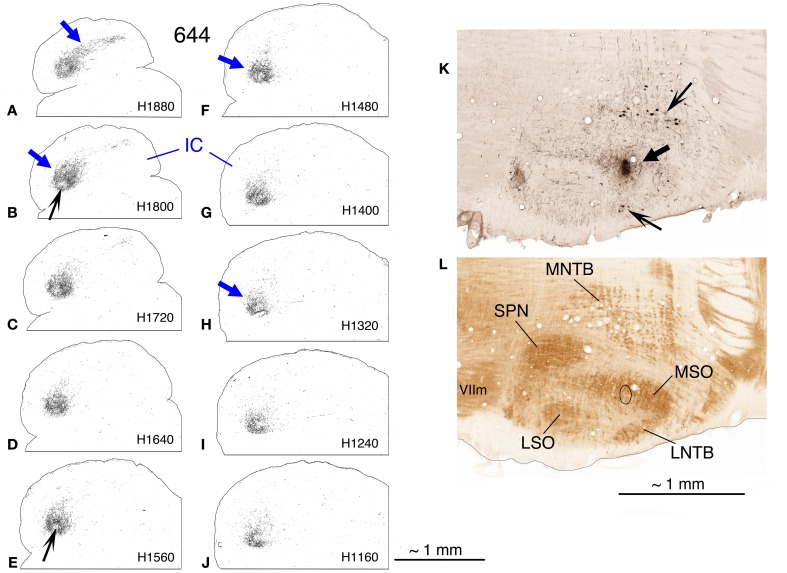
**Case 644, BDA injection in right MSO. (A–J)** Photoshop drawings of evenly-spaced horizontal sections through the top half of the right (ipsilateral) IC. The most dorsal section is illustrated in panel **(A)**; the most ventral, in panel **(J)**. The caudal aspect of the IC is oriented toward the top of each section; the lateral direction is toward the left, and the midline is at the right. Corresponding atlas levels are indicated on each section **(K,L)**. Horizontal sections through the right superior olivary complex. A BDA-reacted section through the MSO is shown in panel **(K)**. The large arrow indicates the injection site; the smaller arrows indicate labeled cells in the MNTB (upper arrow) and LNTB (lower arrow). The ventrally adjacent CO-reacted section is shown in panel **(L)**. Abbreviations: LNTB, lateral nucleus of the trapezoid body; LSO, lateral superior olivary nucleus; MNTB, medial nucleus of the trapezoid body; MSO, medial superior olivary nucleus; SPN, superior paraolivary nucleus; VIIm, motor nucleus of the seventh nerve. Scale bar next to panel **(J)** applies to panels **(A–J)**; scale bar in panel **(L)** applies to panels **(K)** and **(L)**.

**Figure 6:**
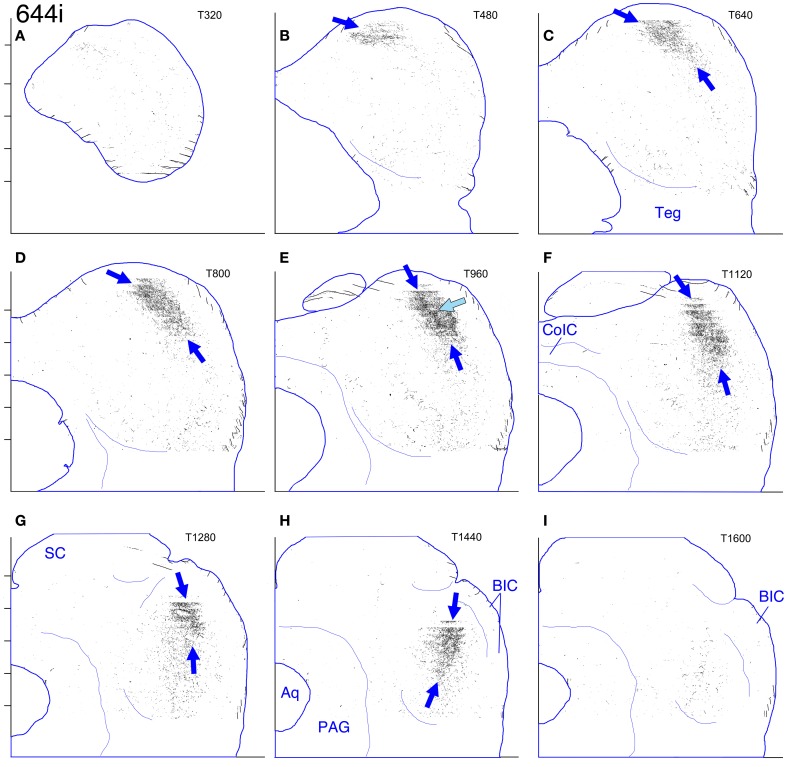
**Case 644i. (A–I)** Reconstruction of the ipsilateral (right) IC after a BDA injection in the dorsal half of the right MSO. In this and also in Figures 9–12, the main plexus of labeled axon terminals is indicated by the blue arrows positioned at its dorsal and ventral extremes. Details as for Figure 2.

**Figure 7:**
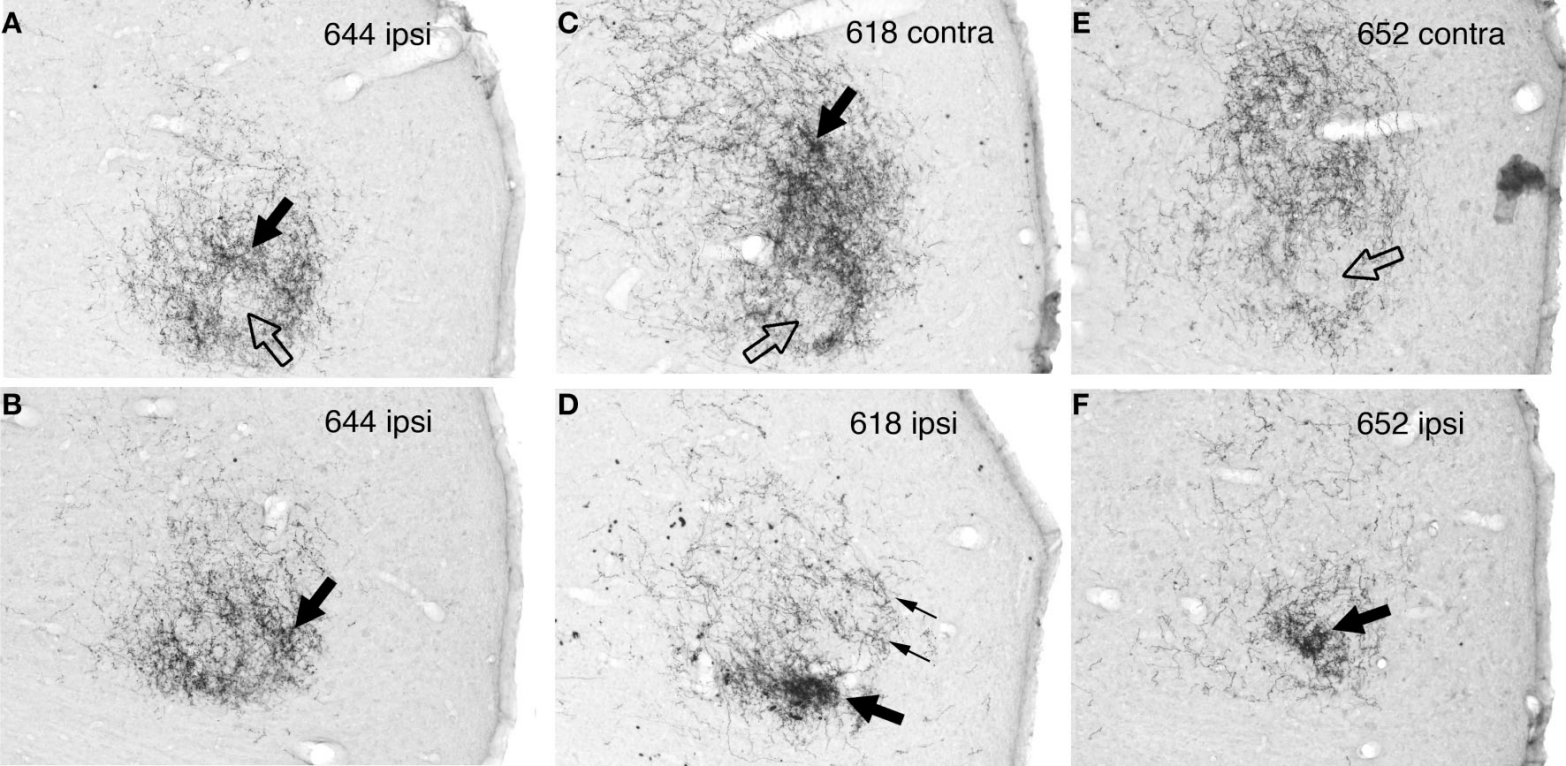
**Digital photographs of the rostrolateral IC in BDA-reacted sections from case 644 (MSO injection) and cases 618 and 652 (LSO injections). (A)** Case 644, ipsilateral IC at level H1720 (also illustrated in Figure 5M). **(B)** Case 644, ipsilateral IC at level H1400 (also illustrated in Figure 5Q). **(C)** Case 618, contralateral IC at level H1400 (also illustrated in Figure 8B). **(D)** Case 618, ipsilateral IC at level H1400 (also illustrated in Figure 8B). **(E)** Case 652, contralateral IC at level H1240. **(F)** Case 652, ipsilateral IC at level H1240. On all panels, the filled black arrows indicate regions of relatively dense terminal labeling, and open arrows indicate areas of relatively sparse labeling. In panel **(D)**, thin arrows point to a hint of banding (also indicated on Figure 8B). In all cases, the lateral boundary of the IC is to the right and the rostral boundary is toward the bottom. (Images of the ipsilateral IC were reflected about the midline to facilitate comparisons.) The brightness and contrast of the images were manipulated using the levels function in Adobe Photoshop.

**Figure 8:**
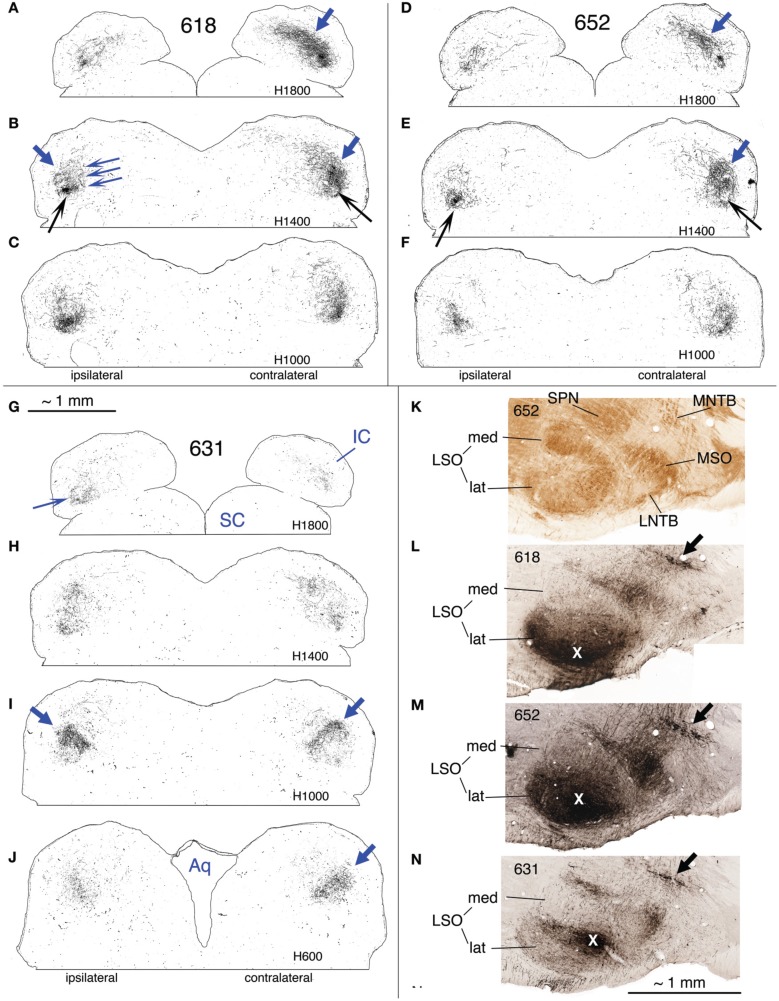
**(A–J)** Photoshop drawings of evenly spaced sections through part of the IC in 3 cases with BDA injections in the right LSO. **(A–C)** Case 652. **(D–F)** Case 618. **(G–J)** Case 631. Scale bar above panel **(G)** applies to panels **(A–J)**. Other details as for Figure 1. **(K–N)** Digital images of horizontal sections through the right superior olivary complex that illustrate the locations of the injection sites in the three cases **(K)**. CO-reacted section from case 652. (The section is ventrally adjacent to that illustrated in panel **(M)**. The sections illustrated in panels **(L)** and **(N)** were located at an equivalent level.) Abbreviations: LNTB, lateral nucleus of the trapezoid body; LSO lat, lateral limb of the lateral superior olivary nucleus; LSO med, medial limb of the lateral superior olivary nucleus; MNTB, medial nucleus of the trapezoid body; MSO, medial superior olivary nucleus; SPN, superior paraolivary nucleus. **(L)** BDA-reacted section through the injection site in case 618. **(M)** BDA-reacted section through the injection site in case 652. **(N)** BDA-reacted section through the injection site in case 631. In panels **(L–N)**, the white “X” indicates the approximate location of the center of the injection site in the LSO. The black arrows indicate rows of labeled cell bodies in the MNTB. Scale bar in panel **(N)** applies to panels **(K–N)**.

**Figure 9:**
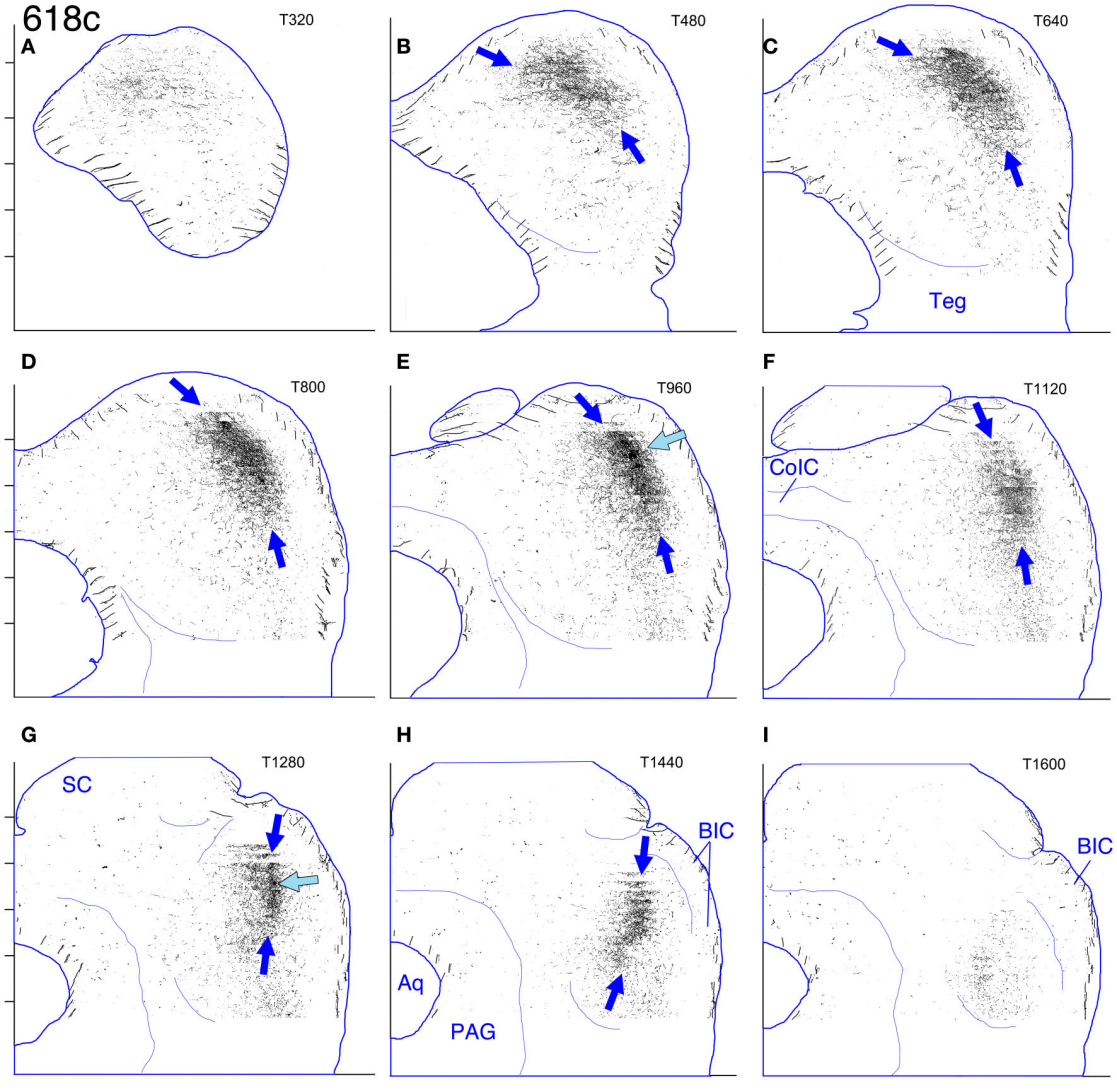
**Case 618c. (A–I)** Reconstruction of the contralateral (left) IC after BDA injection into the right lateral LSO. The sections have been reflected about the midline.

**Figure 10:**
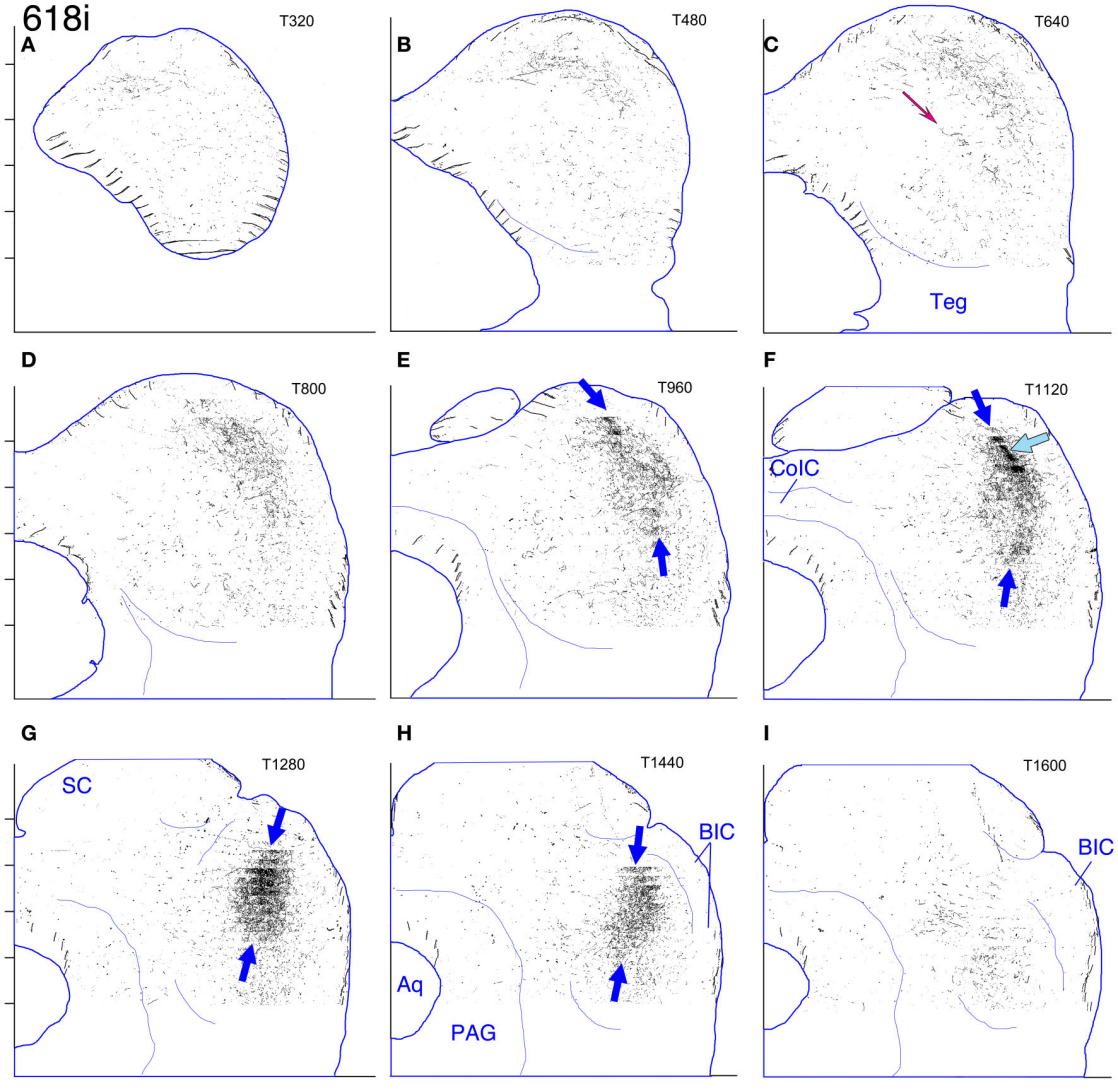
**Case 618i. (A–I)** Reconstruction of the ipsilateral (right) IC after BDA injection into the right lateral LSO.

**Figure 11:**
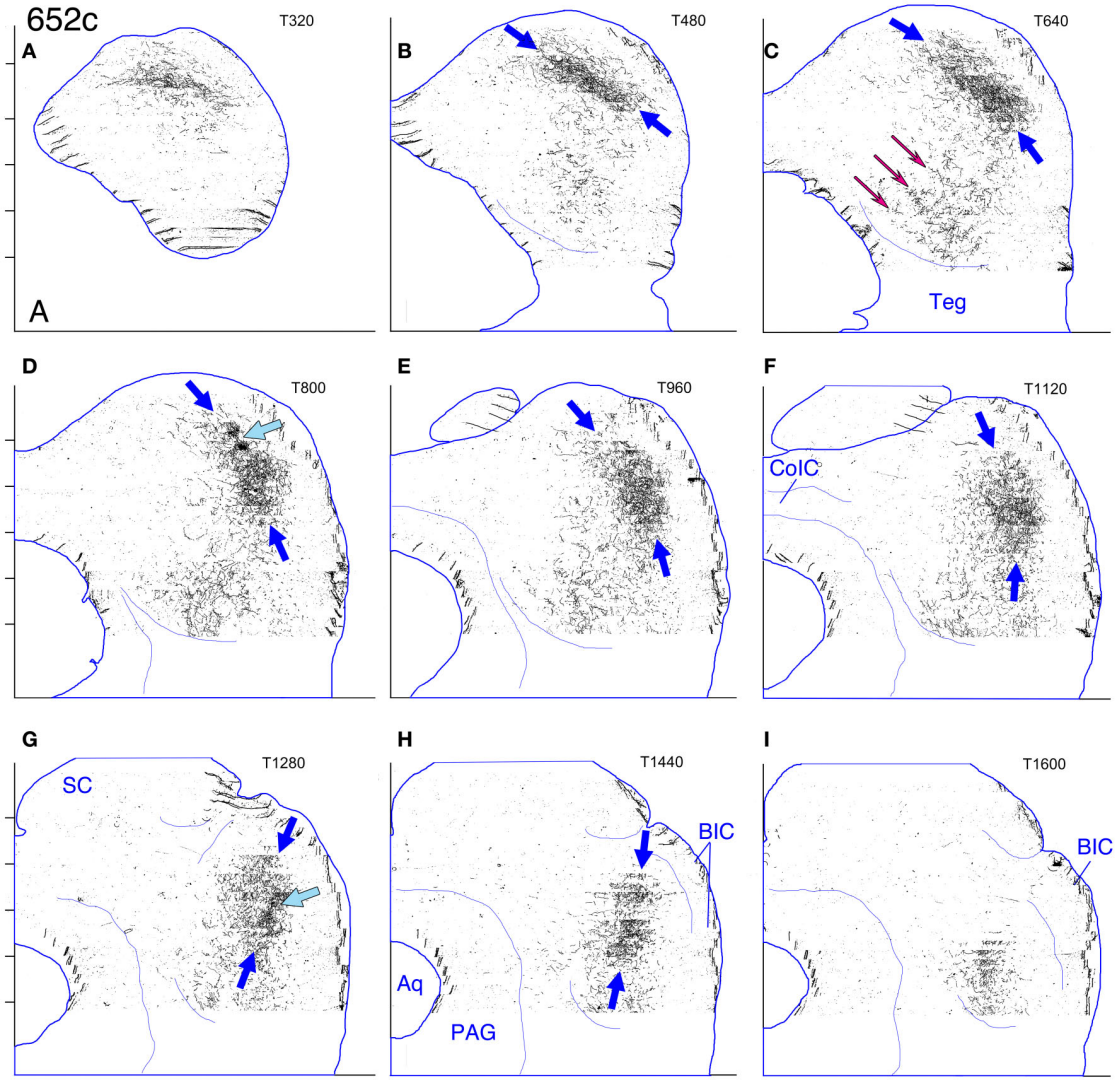
**Case 652c. (A–I)** Reconstruction of the contralateral (left) IC after BDA injection into the right lateral LSO. The sections have been reflected about the midline.

**Figure 12:**
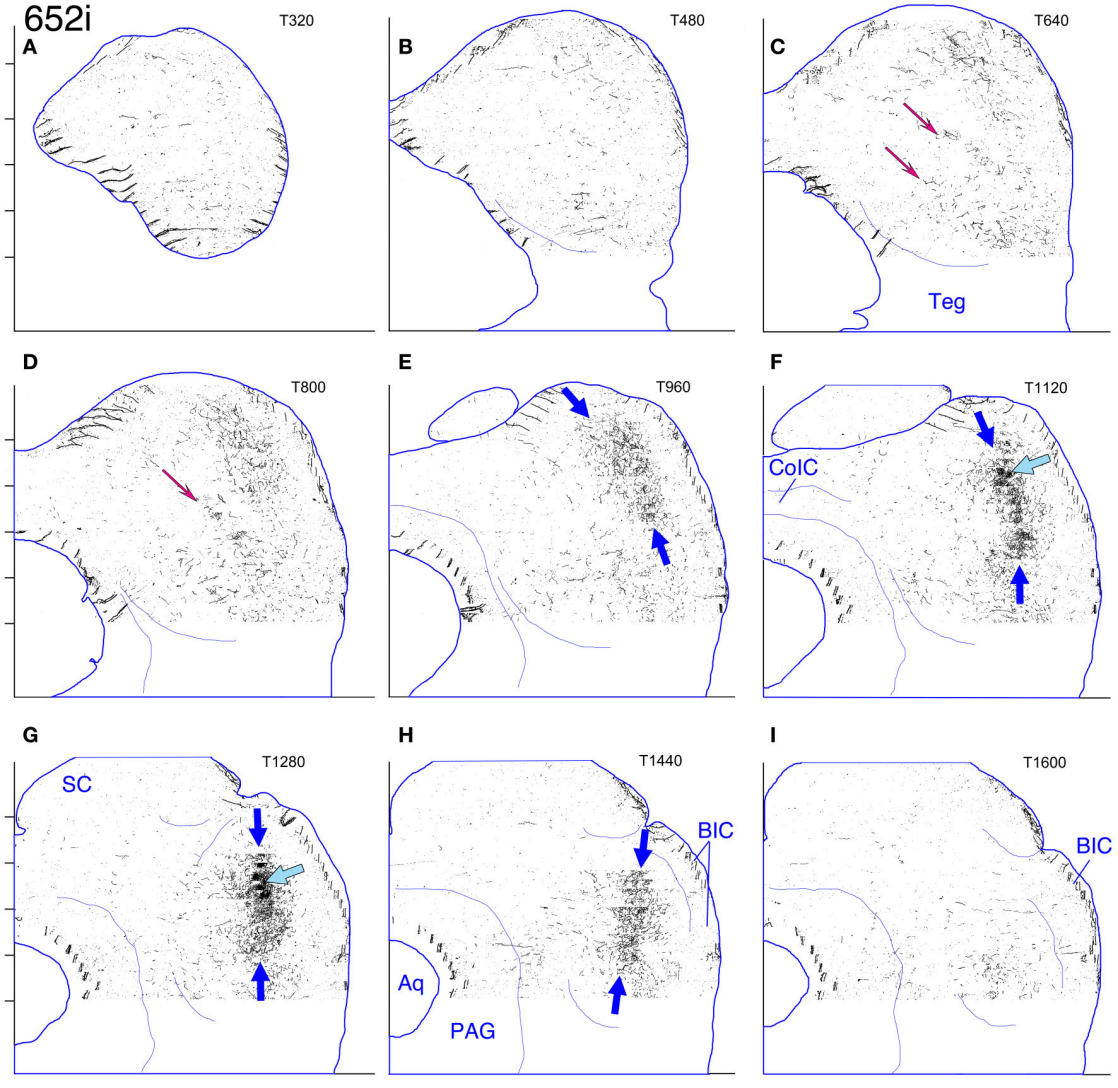
**Case 652i. (A–I)** Reconstruction of the ipsilateral (right) IC after BDA injection into the right lateral LSO.

**Figure 13:**
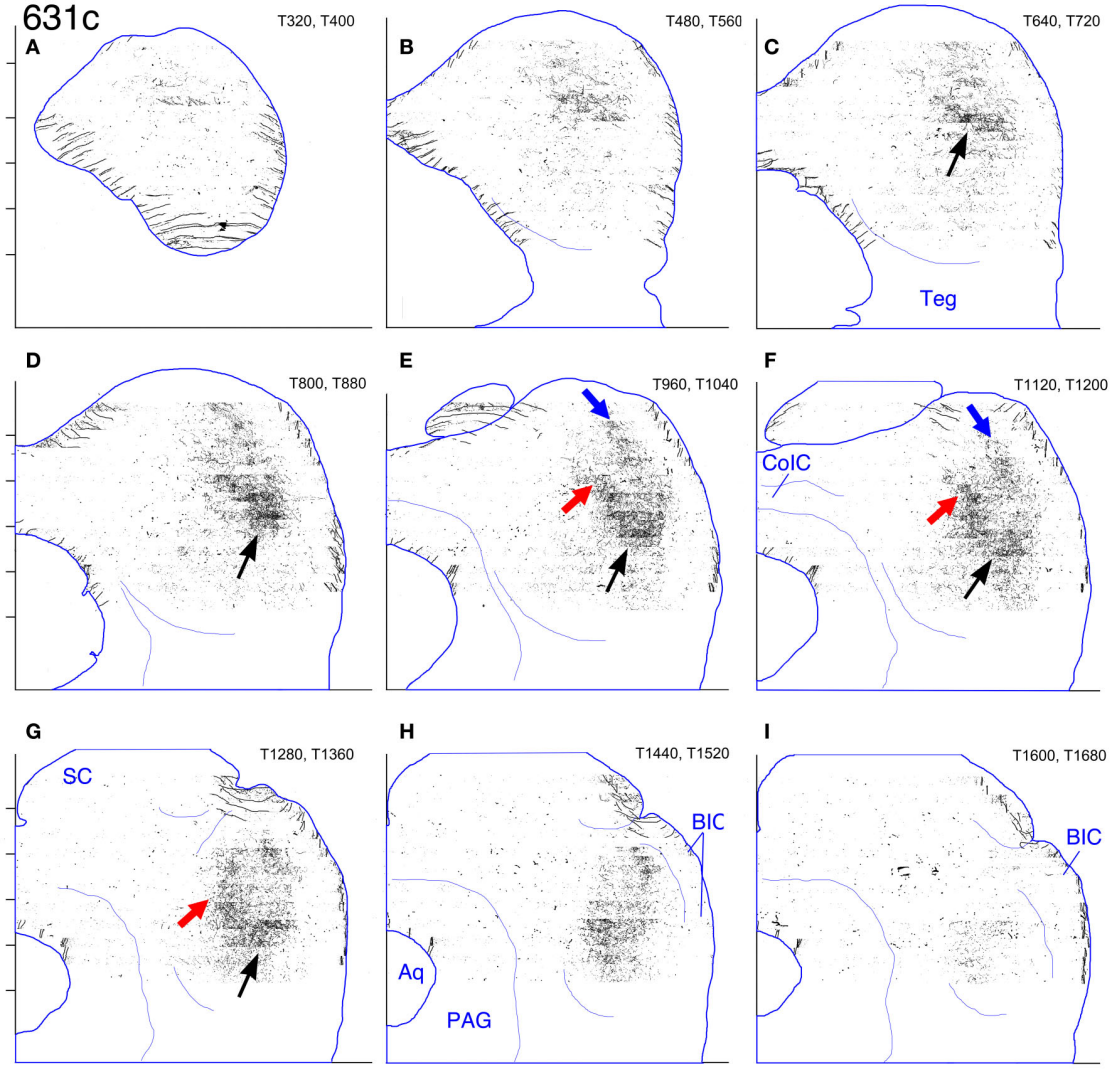
**Case 631c. (A–I)** Reconstruction of the contralateral (left) IC after BDA injection into the right middle LSO. The sections have been reflected about the midline. In this and also in Figure 14, the black and red arrows indicate the most heavily labeled axon plexus (referenced further in the text). The blue arrows indicate a more lightly labeled (and more dorsolaterally located) plexus. For this case only, each panel in the figures represents an overlay of the labeled elements from two transverse sections as indicated in the upper right corner of each. Other details as in Figure 2.

**Figure 14:**
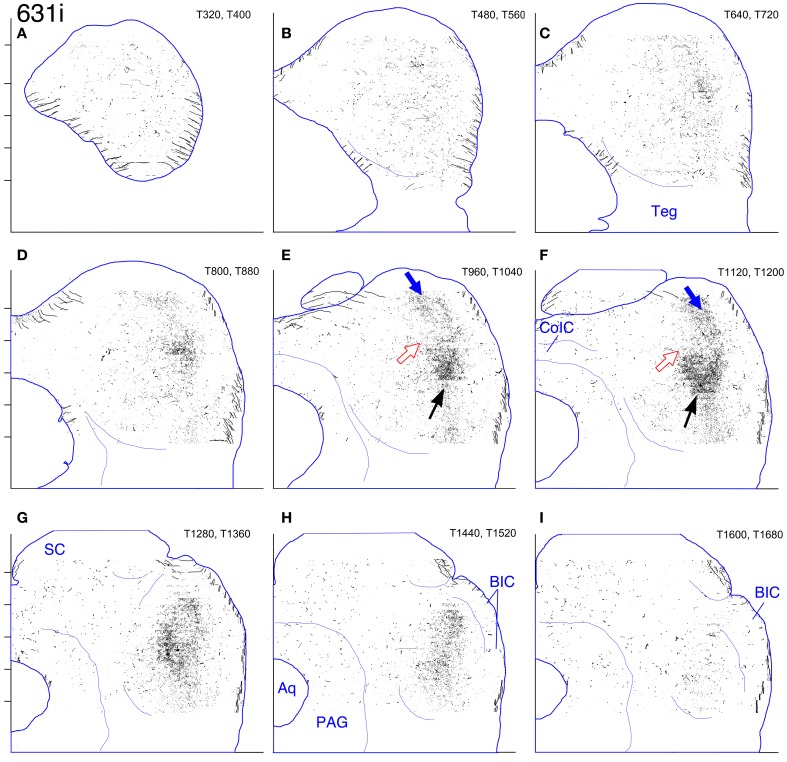
**Case 631i. (A–I)** Reconstruction of the ipsilateral (right) IC after BDA injection into the right middle LSO.

One case with a BDA injection in the MSO and three cases with injections in the LSO are presented in Figures 5–14. Two of the LSO injections were located in the lateral limb. (Both are included here in order to emphasize the consistency of the results.) The third LSO injection was centered in the middle of the nucleus. These four cases illustrate the pattern of olivary projections from the dorsal MSO and the lateral and middle parts of both the ipsilateral and contralateral LSO (i.e., the parts of these olivary nuclei that represent low and, to some extent, middle frequencies).

#### Case 644 (Figures 5, 6, 7A,B)

In the horizontal plane, the injection site in this case (Figure 5K) appears quite small, although it appeared to be elongated in the dorsal to ventral dimension (along the track of the injection pipette, not shown) and was visible in approximately the dorsal 50% of the MSO. Retrogradely labeled cells in the cochlear nucleus were confined to the most rostral part of the anteroventral cochlear nucleus (i.e., the spherical cell area) on both sides and were most numerous ventrally, although labeled cells were present in approximately the lower ¾ of the dorsal-to-ventral extent of the nucleus. In horizontal sections through the dorsal half of the ipsilateral IC, a labeled plexus of axons is located in pars lateralis of the central nucleus (Figures 5A,B,F,H, blue arrows). (A *very* few axon terminals were labeled in comparable sections through the contralateral IC in this case. Their position mirrored that of the most rostrally located axons on the ipsilateral side.) In the most dorsal sections through the ipsilateral IC, the plexus of labeled terminals extends caudomedially in an elongated strip (Figure 5A, blue arrow). In transverse reconstructions (Figure 6), the plexus of labeled axons and terminals has the appearance of an elongated column (Figures 6B–H, bounded dorsally and ventrally by dark blue arrows) that extends from a dorsal position caudally (Figure 6B) to a relatively ventral position rostrally (Figure 6H). Comparison of the appearance of the plexus in horizontal and transverse sections suggests the shape of a long, bent cylinder stretching from the rostroventral boundary of the IC to its dorsocaudal pole.

The plexus of labeled axons appears patchy, containing areas with relatively dense accumulations of terminals (Figure 6E, light blue arrow; Figures 7A,B, black arrows) as well as areas of relatively sparse terminal labeling (Figures 5B,E, black arrows; Figure 7A, open arrow). At some transverse levels, a thin “line” of densely clustered terminals appears to run through the center of the plexus (e.g, Figure 6E, light blue arrow).

#### Cases 618 and 652 (Figures 7C–F, 8A–F, and K–M, 9–12)

These two cases are considered together because the location of the injection sites and the patterns of labeling are very similar. In case 618, the injection site was mostly confined to the lateral limb of the LSO (Figure 8L). Ventrally, however, the injection extended slightly into the rostral part of the lateral nucleus of the trapezoid body (LNTB) and the trapezoid body fibers that run around and through it. Retrogradely labeled cells in the ipsilateral medial nucleus of the trapezoid body (MNTB) were lined up on the lateral boundary along its entire caudal-to-rostral extent (e.g., Figure 8L). In the cochlear nuclei, labeled cells were concentrated in the spherical bushy cell area on both sides and were especially numerous rostrolaterally and ventrally. In addition, on the ipsilateral side only, scattered multipolar cells were distributed throughout the ventral cochlear nucleus. A very few small cells were labeled in the ipsilateral dorsal cochlear nucleus. The injection site in case 652 (Figure 8M) was located in almost exactly the same part of the LSO as that in case 618, but did not appear to extend into the LNTB. The locations of labeled cells in the ipsilateral MNTB (e.g., Figure 8M) and in the ipsilateral and contralateral ventral cochlear nuclei followed the same pattern as in case 618, although the number of labeled cells was greater. One potential complication in case 652 (and in case 631, presented below) is that the injection pipette passed through the IC itself on the way to the LSO. Its track can be seen as a small spot of decreased cytochrome oxdase activity on sections through the IC (cf. Cant and Benson, 2006). No anterograde labeling of either cells or axons appeared to be associated with this track.

The terminal plexus in the *contralateral* IC in both cases (618: Figures 8A–C and 9; 652: Figures 8D–F and 11) occupies pars lateralis of the central nucleus. The location of the plexus is similar to that seen after the MSO injection (case 644, Figures 5A–J, 6) except that the dorsal-to-ventral position of the plexus in case 618 appears to be shifted slightly ventrally relative to that in case 644, and that in 652 appears to be shifted slightly ventrally relative to that in 618. In the most dorsal sections, the labeled plexus extends across the width of the IC (Figures 8A,D, blue arrows). The labeled plexus in the *ipsilatera*l IC in these cases (618: Figures 8A–C and 10; 652: Figures 8D–F and 12) is in a location similar to that on the contralateral side but is less extensive, being almost absent at caudal levels (compare Figures 9A–C to Figures 10A–C and Figures 11A–C to Figures 12A–C).

On both the ipsilateral and contralateral sides, the appearance of the terminal plexus is patchy. Small areas containing dense tangles of axon terminals (e.g., Figures 7C,D,F, black arrows; Figures 9E,G; 10F; 11D,G; 12F, light blue arrows) are interspersed among areas of relatively sparser terminations (e.g., Figures 7C,E, open arrows). At some levels an ipsilateral dense patch appears to occupy a position comparable to the location of a relatively empty space on the contralateral side (e.g., Figure 7C compared to Figure 7D and Figure 7E compared to Figure 7F; similar comparisons can be made of Figures 8B and 8E, thin black arrows).

On the contralateral side in case 652 (and to a lesser extent on the ipsilateral side) there is sparse but definite terminal labeling in the ventral (i.e., high frequency) part of the IC (Figures 11, 12). Caudally, the ventral axons appear to form interrupted stacks (Figures 11C and 12C, thin magenta arrows), reminiscent of the banding of LSO axons described by others (see “Discussion”). Banding of inputs is less obvious in the more densely labeled dorsolateral terminal field, although there is sometimes a hint of it (e.g., Figure 7D, thin arrows; Figure 8E, ipsi, thin blue arrows). In case 618, which otherwise exhibits almost the same labeling pattern as that in case 652, axons in the ventral IC are not labeled (with the exception of one tiny tuft on the ipsilateral side at atlas level T640, Figure 10C, magenta arrow).

#### Case 631 (Figures 8G–J,N, 13, 14)

The apparent injection site in this case (Figure 8N) was considerably smaller than those in cases 618 and 652. (It was more comparable in size to that in case 644). It appeared to be mostly confined to the middle of the LSO. In the transverse plane, the LSO in the gerbil has the shape of a baby duck; the injection site was located approximately at the duck's neck. According to maps of the adult gerbil LSO constructed by Sanes et al. (1989), the frequency representation in this part of the IC would be around 3–6 kHz. Ventrally, the injection site appeared to encroach slightly on the lateral part of the LSO. As noted above, the pipette in this case passed through the IC on its way to the LSO, but no labeled axons or terminals were visible around the location of the track. Retrogradely labeled cells in the MNTB (e.g., Figure 8N) stretched along its caudal-to-rostral (and dorsal-to-ventral) extent and the sheet of cells was centered at about 30–35% of its lateral-to-medial extent. As with cases 618 and 652, labeled cells were plentiful in the spherical bushy cell area in the anteroventral cochlear nucleus on both sides but were shifted dorsomedially with respect to those cases. In addition, scattered cells (most likely, multipolar cells) were located throughout the ipsilateral ventral cochlear nucleus.

In the IC on both sides, a labeled terminal plexus lies in the ventral part of pars lateralis (Figures 8I–J, blue arrows; Figures 13C–G, black arrows; Figures 14E,F, black arrows) and also extends out into the territory of the main intrinsic plexus described above (Figures 12E–G, red arrows), where it forms a truncated layer with a ventrolateral to dorsomedial tilt. The axonal plexus in the ipsilateral IC is less widely distributed than that on the contralateral side, especially at caudal levels (compare Figures 13B–D [contralateral] to Figures 14B–D [ipsilateral]). The extension of the plexus outside pars lateralis also appears to be truncated compared to that on the contralateral side. For example, the same point with respect to the atlas coordinates is indicated by the red arrows in Figures 13E,F (contralateral side) and the open red arrows in Figures 14E,F (ipsilateral side). In general, the labeled plexus in case 631 appears to be less patchy than those in cases 644, 618, and 652.

On both the ipsilateral and contralateral sides in case 631, in addition to the heavily labeled plexus located in the middle of the IC, a less dense axonal plexus occupies the same position in the pars lateralis seen for the plexuses in cases 644, 618, and 652 (Figure 8G, thin blue arrow; Figures 13E–F, 14E–F, blue arrows). The presence of this lightly labeled plexus is most likely accounted for by the fact that the injection site, although mainly located in the middle of the LSO, probably also encroached on the lateral LSO.

#### Comparisons among cases

**Figure 15:**
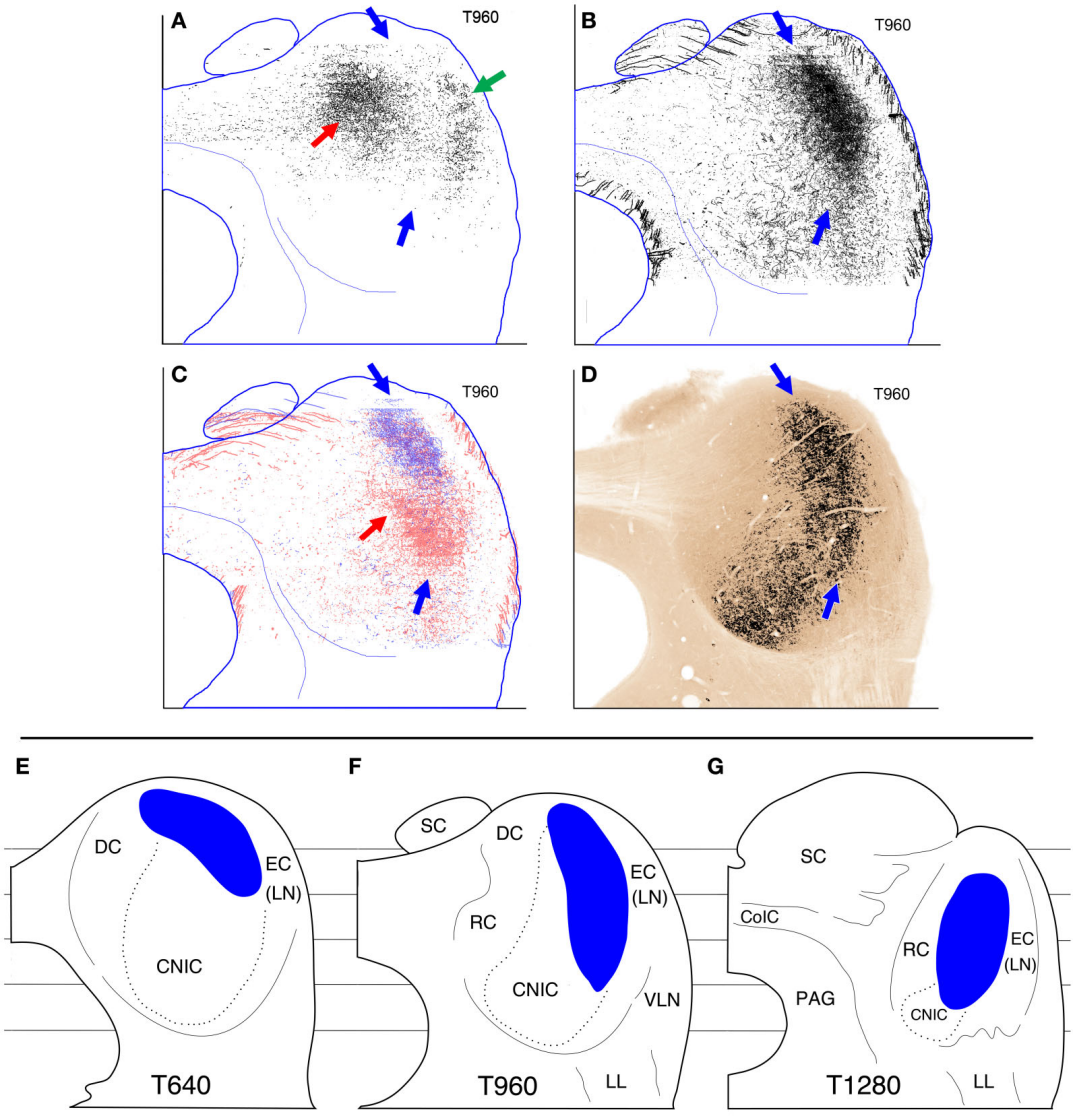
**(A–C)** Comparisons of anterograde labeling patterns at one transverse atlas level (T960). **(A)** Overlap between the labeled plexuses in cases 460 and 462 (IC injections, Figures 2E and 3E). The black fill indicates the pixels that were filled at this atlas level in *both* cases. There was substantial overlap in the main plexus (red arrow) and the lateral plexus (green arrow) but very little overlap in pars lateralis (dorsal and ventral extent indicated by blue arrows). **(B)** Overlay of the reconstructions at level T960 from the ipsilateral and contralateral IC in cases 618 and 652 (injections in lateral limb of LSO, Figures 9–12, panel **E**). Most of the labeled terminals and axons in these 4 ICs overlap in pars lateralis (blue arrows). **(C)** Overlay of the reconstructions at level T960 from the ipsilateral MSO in case 644 (from Figure 6E, blue pixels) and the contralateral LSO in case 631 (injection in middle LSO; from Figure 12E, red pixels). Most of the labeled terminals from both cases lie in pars lateralis, but some terminals in case 631 also extend into a more medial part of the central nucleus (red arrow) **(D)**. Cytochrome oxidase-reacted section at atlas level T960. The part of the IC with the highest CO activity is highlighted in black (see Cant and Benson, 2005, 2006). The blue arrows indicate the approximate dorsal and ventral boundaries of the CO-dense area identified as pars lateralis **(E–G)**. Schematic drawings of the gerbil IC at three transverse levels: **(E)** T640, **(F)** T960, **(G)** T1280. The blue fill indicates the approximate spatial extent of the pars lateralis of the central nucleus in the gerbil as described in the text. The dotted outline indicates the approximate boundaries of the rest of the central nucleus. For each drawing, dorsal is toward the top and lateral is toward the right. The lines behind the outlines of the sections indicate, from top (dorsal) to bottom (ventral), the levels of horizontal atlas sections H1800, H1400, H1000, H600, and H200. Abbreviations: CNIC, central nucleus of the inferior colliculus; CoIC, commissure of the inferior colliculus; DC, dorsal cortex; EC, external cortex; LL, lateral lemniscus; LN, lateral nucleus; PAG, periaqueductal gray matter; RC, rostral cortex; SC, superior colliculus; VLN, ventrolateral nucleus.

Because all of the cases presented here were mapped onto a common set of coordinates, direct comparisons of labeling patterns can be made at different levels through the IC. As examples, a few of the possible comparisons at one transverse level are presented in Figures 15A–C. These particular comparisons were chosen to support the interpretation of the results developed in the Discussion; they are representative of the results generally. Figure 15A illustrates the region in which there was overlap of the terminal plexuses in cases 460 and 462 (injections in contralateral IC) at transverse level T960 (Figures 2E and 3E, respectively). The black fill in this panel represents *only* those filled pixels which were common to both of the cases. The important point is that there is considerable overlap in the region of the main terminal plexus (red arrow) and in the region of the lateral terminal plexus (green arrow) but that very little overlap is evident between these two plexuses (that is, in the pars lateralis, blue arrows). Reference to Figures 2D,E and 3D,E (black arrows) confirms that labeled axon terminals were located in pars lateralis in both of these cases at this level; however, those in case 462 were located more dorsally than those in case 460 with the result that there was very little overlap. In Figure 15B, the labeled terminal plexuses at level T960 from both sides in cases 618 and 652 (injections in lateral LSO) are superimposed. The shape and general location of the pixels from the four ICs combined (blue arrows) looks very similar to those in each individual IC (i.e., Figures 9–12, T960, blue arrows). Figure 15C illustrates the plexuses labeled in case 644 (MSO injection, blue pixels) and case 631 (middle LSO injection, red pixels). As for cases 618 and 652 (Figure 15B), the labeled axons in case 644 and most of those in case 631 lie in pars lateralis (blue arrows), but in case 631, a small extension into the central part of the central nucleus is also present (red arrow). An important point is that the plexus in case 631 lies *ventral* to that in case 644 (and also to those in cases 618 and 652, compare Figure 15C to Figure 15B). Figure 15D illustrates the cytochrome-oxidase stained atlas section at level T960. The part of the IC with the highest CO activity is highlighted in black. As described previously (Cant and Benson, 2005), this region of highest metabolic capacity forms a crescent-shaped swath through the IC at middle levels (as in this figure). The blue arrows indicate the dorsal and ventral extent of the part of this crescent that I have referred to as pars lateralis of the central nucleus.

## Discussion

The results provide new information about the organization of the part of the IC that receives inputs from the LSO and MSO in the gerbil. The discussion of the results is divided into three parts. First, interpretation of the injection sites is examined. Second, a working model of the basic organization of the gerbil IC is presented and compared to the more well-established models for the rat and cat. Finally, I discuss the organization of one subdivision of the central nucleus of the IC—the pars lateralis—in these three species.

### Interpretation of the injection sites

BDA is an excellent anterograde tracer, but interpretation of results is complicated by the fact that it can be transported to terminal fields arising from axonal branches of neurons located outside the injection site that send a separate branch into the site (so-called “false anterograde” or “collateral” label; Chen and Aston-Jones, 1998; see Discussion in Saldaña et al., 2009). Because of this, the more that is known about the branching and projection patterns of the labeled neurons, the more convincing the interpretation of the origin of any particular terminal field can be.

The injection sites for the three cases with BDA in the IC itself (Figures 1–4) were previously discussed in Cant and Benson (2006). The arguments presented in that paper did not depend on a precise definition of the effective uptake area in each case (the center of the site being the most important consideration), nor does it make much difference for the arguments presented here. The sources of labeling after an injection into one IC have been discussed in detail elsewhere (e.g., Saldaña and Merchán, 1992; Malmierca et al., 2009). What is important for the purposes of the present study is the shape and arrangement of the terminal plexuses that are labeled, regardless of the source of the neurons that give rise to them.

On the other hand, it is critically important to consider the possible sources of terminals in the cases with injection sites in the MSO or LSO. In all of these cases, spherical bushy cells were labeled in the ventral cochlear nucleus on both sides, principal cells were labeled in the ipsilateral MNTB, and cells were labeled in the ipsilateral LNTB, all well-known sources of input to the MSO and LSO (reviewed by Schofield, 2005). Neither the bushy cells nor the MNTB principal cells project to the IC (Cant and Benson, 2003; Schofield, 2005). The LNTB, however, does project bilaterally to the IC (Schofield and Cant, 1992; Schofield, 2005), and it is not known whether the LNTB cells that project to the MSO or LSO also project to the IC. Therefore, the LNTB must be considered as a potential source of some of the terminals labeled in all of the SOC-injection cases, especially in case 618 in which the injection site probably included a portion of the LNTB. Although it seems most likely that terminals contributed by the LNTB would be substantially fewer than those contributed by the MSO or LSO (where the injection sites are centered), to my knowledge, this has not been demonstrated experimentally.

In the ipsilateral cochlear nuclei in the cases with injection sites in the LSO, multipolar cells are also labeled. A projection to the LSO from multipolar cells is well-documented (e.g., Doucet and Ryugo, 2003), and it appears to arise partly or exclusively from the “planar” multipolar cells (or “T-stellate” cells; see Oertel et al., 2011). Planar multipolar cells also project to the IC (reviewed in Cant and Benson, 2003; Oertel et al., 2011), and although it is not known whether the same neurons give rise to both projections, it is possible that they do and that some of the anterogradely labeled axons in the cases described here arise from the ventral cochlear nucleus rather than from the LSO. However, it seems highly unlikely that LSO-projecting multipolar cells are a major source of the labeled plexuses in the IC for several reasons. First, cells in the cochlear nucleus labeled after IC injections (e.g., Cant and Benson, 2006) are much more numerous and, in general, more densely packed than those in the LSO injection cases described here, in which they are sparsely distributed. Second, in IC injection cases (e.g., those described in Cant and Benson, 2006), in which multipolar cells are labeled in the VCN, there is only sparse terminal labeling (if any) in the LSO on either side, even in cases with very large numbers of labeled multipolar cells in the VCN. This is evidence (albeit negative) that the multipolar cells that project to the IC do not also project to the LSO. Finally, Doucet and Ryugo (2003) noted that the main (or perhaps only) projections from multipolar cells to the LSO were to its middle and high frequency parts; they did not see terminations in the lateral (low frequency) limb of the LSO. Thus, injections in the part of the LSO described in the present paper may be less likely to include any terminations from multipolar cells that do happen to project to both the IC and LSO. In summary, both the LNTB and VCN could be sources of some of the terminal labeling described in the LSO injection cases described in this report, but they are not likely to account for a substantial proportion of that labeling.

One mystery that I do not have a good solution for is the source of the relatively sparse terminations in the *ventral* IC in case 652. The injection sites in the LSO in cases 652 and 618 appear very similar, but only case 652 exhibits this labeling in the part of the IC that represents high frequencies. Although present on both sides, the ventral labeling is most prominent on the ipsilateral side, which would seem to rule out the VCN as a source since, in the gerbil, the ipsilateral projections from the VCN terminate almost exclusively in the dorsal and rostral IC (Nordeen et al., 1983; Cant and Benson, 2008). One possibility is that they are from the LSO itself. The banded pattern certainly fits with this possibility (see later Discussion), but the axons that leave the high frequency part of the LSO do not pass through the low frequency part (where the injection is; unpublished observations).

### Comparison of the IC in gerbil to the IC in rat and cat

Loftus et al. (2008) argued that superficial differences in the appearance of the IC among species may be the result of a difference in the proportion of collicular space devoted to a particular functional region rather than to a fundamental difference in the basic plan of organization. In the gerbil, a large proportion of the IC appears to be devoted to frequencies below about 3 kHz (e.g., Ryan et al., 1982; Harris et al., 1997), and the representation of middle and higher frequencies is consequently relatively compressed. Allowing for this difference, the basic plan of the gerbil IC appears to be like that of the cat and rat. To emphasize important commonalities, I have used the nomenclature developed in the rat and cat for the model presented below.

The present results, in combination with those previously reported (Cant and Benson, 2006, 2007, 2008), form the basis for the working model of the organization of the gerbil IC presented in Figure 15, panels E–G. Schematic representations at caudal (T640), middle (T960), and rostral (T1280) levels (Figures 15E–G, respectively) illustrate the general appearance of the subdivisions. The central nucleus (which is roughly defined according to Cant and Benson, 2005, 2006) is itself divided into two parts: a relatively dorsolateral and rostral part referred to as pars lateralis (Figures 15E–G, blue fill) and a relatively ventromedial and caudal part (bounded by a dotted line) that itself can probably be further subdivided (see below). Surrounding these two parts of the central nucleus are a dorsal cortex, a rostral cortex, an external cortex, (or lateral nucleus) and a ventrolateral nucleus. I have not attempted to illustrate boundaries between these surrounding (or shell) regions; their more precise delineation is a goal of continuing studies.

With the caveat that the relative volume devoted to different frequency ranges is markedly different, the auditory midbrain in rats and gerbils appears to be organized in much the same way. Faye-Lund and Osen (1985) provided a detailed and systematic description of the rat IC. With several modifications proposed by others and one new modification suggested below, their parcellation applies to the gerbil as well. First, a rostral and medial part of the IC that was included in the external cortex of Faye-Lund and Osen (1985) is now recognized as a separate subdivision and is referred to as the rostral cortex (e.g., Saldaña and Merchán, 2005; Malmierca et al., 2011). This designation is also appropriate for the gerbil. A second modification of the original scheme for the rat is based on comparisons to the cat. Loftus et al. (2008) noted that the external cortex along the lateral surface of the rat IC is considerably thicker and more obviously laminar in the rat compared to the cat. Their explanation for the difference seems to apply to the gerbil as well. That is, with a relative expansion of the low frequency representation in the cat compared to that in the rat, the prominent third layer characteristic of the rat external cortex (which they renamed the ventrolateral nucleus) becomes displaced ventrally, where it forms a smaller ventrolateral nucleus that is considered to be equivalent to the larger and more extensive version in the rat. A ventrolateral nucleus can also be identified in the gerbil (Cant and Benson, 2008).

I suggest one further modification to the rat scheme that involves the delineation of the central nucleus. In the gerbil, the central nucleus, if defined as the part of the IC with the highest relative CO activity (Cant and Benson, 2005, 2006), extends almost all the way to the rostral and dorsal boundaries of the IC. Unlike the location of the central nucleus as defined in the rat, it is not confined to either the medial 2/3 or the caudal 2/3 of the IC and, in the lateral part of the IC, is not flattened in the frontal plane. I suggest that the apparent difference between the rat and gerbil can be reconciled by reconsidering the identification of the rostrolateral area that Faye-Lund and Osen (1985) called the “lemniscal field” (and that they labeled LL, “like the lemniscus itself”). Although “lemniscal field” may be an apt description (this part of the IC is relatively heavily myelinated and axons arising from the lemniscus do extend into this part of the central nucleus—*as well as into other parts*), the choice of the label “LL” implies that the area represents a dorsal continuation of the fiber bundle itself (although it is clear from their figures that this is not the case). In fact, the dorsolateral IC (the “lemniscal field”) is filled with neurons and terminals. In contrast to the lateral lemniscus itself, which, like all fiber bundles, exhibits relatively low CO activity, this part of the IC exhibits CO activity as high or higher than any other part of the structure (e.g., gerbil: Gonzalez-Lima and Jones, 1994; Cant and Benson, 2005; rat: Loftus et al., 2008; mouse: Gonzalez-Lima and Cada, 1994).

A good agreement between the appearance of the central nucleus in gerbils and rats is achieved if the rat's lemniscal field is incorporated into its central nucleus. A direct comparison can be made between Figure 1 in the present report and the schematic diagram in Figure 11 of Faye-Lund and Osen (1985). The blue arrows in Figure 1 (panels **A–C** and **G,H**) point to the rostral part of the central nucleus in the gerbil; the same relative location is labeled “LL” on the middle panels in Faye-Lund and Osen's Figure 11, column 3. This is the part of the central nucleus in the gerbil that represents low frequencies (Ryan et al., 1982; Brückner and Rübsamen, 1995; Cant and Benson, 2008). The low frequency representation is considerably smaller in the rat (Ryan et al., 1988), but the relative location in the dorsolateral and rostral IC appears to be consistent with the location of the lemniscal field. In contrast to my interpretation as stated here, Gonzalez-Lima and Jones (1994) in their survey of CO activity patterns in the auditory nuclei of the gerbil apparently *excluded* the dorsal and rostral part of the central nucleus as we have defined it and instead, following Faye-Lund and Osen (1985) labeled that region the “lateral lemniscal field” (their Figure 10). Thus, although there is agreement about the correspondence between the lemniscal field in the rat and a part of the gerbil IC that has high CO activity, I have concluded that both should be considered a part of the central nucleus, whereas Gonzalez-Lima and Cada did not. My rationale for considering this to be a part of the central nucleus is that the dorsal and rostral part of the IC is where the lowest frequencies are represented and, as shown in this paper, it is also the terminal zone of the projections from the low frequency parts of the MSO and LSO. If this part of the IC is excluded from the central nucleus, then the central nucleus would not include a part of the structure involved in low-frequency, binaural processing. Further justification for this interpretation is based on comparisons to the cat as discussed in the next section.

Oliver and Morest (1984), in their studies of the cat, were the first to identify subdivisions in the central nucleus of the IC. They defined three main parts based on the arrangement of the fibrodendritic laminae characteristic of the central nucleus in Golgi preparations. Their pars lateralis occupies the lateral and dorsal part of the central nucleus and extends to its rostral boundary where the fibrodendritic laminae assume a curved shape. This is the same relative position occupied by the part of the gerbil central nucleus that I have identified as pars lateralis (Figure 15); the curved arrangement of the laminae is reflected in a curved arrangement of inputs from the ventral parts of the cochlear nuclei (i.e., the fibers representing low frequencies; Cant and Benson, 2008). This part of the central nucleus exhibits high levels of CO activity in both the gerbil (Figure 15D; also Cant and Benson, 2005, 2007) and the cat (Loftus et al., 2008).

The rat central nucleus was not subdivided explicitly by Faye-Lund and Osen (1985), but my conclusion, as discussed above, is that they did actually define a lateral subdivision, that is, the area they called the “lemniscal zone.” I suggest that this small zone is, in fact, analogous to the proportionately much larger pars lateralis in the cat and gerbil. In addition to the similar shape and position in the IC, several other observations support this interpretation. First, similar to the pattern in the gerbil, the dorsolateral and rostral IC in the rat exhibits relatively high CO activity (unpublished observations; a similar region of high metabolic capacity is seen in the dorsolateral IC of the mouse, Gonzalez-Lima and Cada, 1994; Willott, 2001). Also in common with the cat and gerbil, the extreme dorsolateral part of the rat IC represents the lowest frequencies processed by the rat (e.g., Ryan et al., 1988), and is the target of projections from the MSO (Saldaña et al., 2009).

The central nucleus of the IC is usually modeled as a stack of layers representing successively higher frequencies from the top (dorsal) to the bottom (ventral) of the stack (e.g., Merzenich and Reid, 1974). In this sense, the pars lateralis could be taken to represent the top layer(s) in the stack. In the caudal IC (e.g., as schematized in Figure 15E), this description may fit, but moving to middle and rostral levels (Figures 15F,G), this interpretation does not seem appropriate because the pars lateralis, taken as a whole, does not have the shape of a disk-shaped layer at the top of a stack. Rather it takes on the shape of a long bent cylinder that extends upward from the rostroventral boundary of the IC, curving caudally and dorsally and finally extending medially just beneath the dorsal surface of the IC. The same shape is seen in the part of the central nucleus of the cat that is activated by a 500 Hz tone (2-deoxyglucose studies, Brown et al., 1997, their Figure 5A). and, to some extent, in the more dorsal (low frequency) laminar plexus reconstructed in three dimensions in the guinea pig (Malmierca et al., 1995). This apparent divergence from the more orderly stacks that characterize most of the central nucleus could be the reason why Merzenich and Reid (1974) observed that the “series of stacked disks” in the cat appear to be “simultaneously toppling rostrally and laterally.”

### Pars lateralis of the central nucleus is a major target of low-frequency, binaural inputs

Oliver and Morest (1984) raised the possibility that each subdivision of the central nucleus plays a different functional role in auditory processing. The unique neuroanatomical organization of pars lateralis suggests that it is primarily involved in integrating binaural inputs from the superior olivary complex and the cochlear nuclei. Further, the pars lateralis may project to a part of the ventral division of the medial geniculate nucleus separate from the projections of other parts of the central nucleus (Cant and Benson, 2007).

#### Input from the superior olivary complex

Henkel and Spangler (1983) demonstrated that the axons arising from the MSO in the cat terminate in only a part of the central nucleus, and they suggested that the terminal field might be restricted mainly to the pars lateralis. Their results were corroborated by Oliver et al. (1995), who further demonstrated that up to 36% of the excitatory terminals (i.e., terminals with round synaptic vesicles) in pars lateralis originate in the MSO. Indeed, several studies in the cat include cases in which a large majority (up to greater than 90%) of labeled cells are located in the MSO after small injections of a retrograde tracer in the lateral central nucleus (Roth et al., 1978; Brunso-Bechtold et al., 1981; Aitkin and Schuck, 1985; Loftus et al., 2010). In our studies in the gerbil, labeled cells in the MSO were always accompanied by labeled cells in the LSO and cochlear nuclei (Cant and Benson, 2007), but our injections were not as small as those in the studies cited above. The results of the small injections are consistent with the view that the IC laminae are made up of a mosaic of anatomically (and, therefore, functionally) distinct areas (e.g., Oliver and Huerta, 1992; Loftus et al., 2010). (The larger injections most likely include a number of the small areas and so could mask any specificity of connections).

In the cat, terminations from the MSO, even from its ventral-most part, do not extend all the way into the ventral part of the central nucleus (Henkel and Spangler, 1983). The results of our retrograde tracing experiments suggest that the same is true for the gerbil, as the MSO never contained more than a few labeled cells (if any) when the injections were in the ventral IC (Cant and Benson, 2006). In the cat, cells in the ventral MSO may terminate in pars centralis of the central nucleus (suggested on the basis of the patterns illustrated by Henkel and Spangler, 1983, their Figure 11 and by Loftus et al., 2004, their case 56). The pars centralis appears to be proportionately much smaller in the gerbil than in the cat, and it is difficult to come to a conclusion about its input from the MSO in the gerbil. The MSO in the gerbil appears to be heavily biased toward the lower frequency range (i.e., below about 3 kHz) based on patterns of 2-deoxyglucose uptake during tonal stimulation (Ryan et al., 1982), and it is possible that terminations from the MSO in the gerbil are confined entirely to the pars lateralis.

Both our retrograde results (Cant and Benson, 2006) and the anterograde results presented here suggest that the inputs from the ipsilateral and contralateral LSOs overlap extensively with the inputs from the MSO in the pars lateralis as a whole, but that at a local level, the inputs from these sources are not distributed homogeneously. In the two cases with tracer injections in the lateral limb of the LSO (cases 618 and 652), the ipsilateral and contralateral inputs appear to form complementary terminal fields at some (but not all) levels. This is compatible with the findings of Loftus et al. (2004, 2010) in the cat that the inputs from the ipsilateral MSO and LSO appear to overlap locally in parts of the central nucleus that do not receive input from the contrateral LSO. (It is not known whether the excitatory and inhibitory projections from the ipsilateral LSO [e.g., Saint Marie et al., 1989] are distributed differentially.) Like the MSO, both LSOs contribute a significant number of the excitatory inputs to the parts of the central nucleus in which they terminate. Oliver et al. (1995; Oliver, 2000) estimated that the contralateral LSO contributes up to 18% of the excitatory terminals in some parts of the central nucleus and that the ipsilateral LSO can contribute up to 26% of the excitatory terminals. However, given the results of Loftus et al. (2010; also the present results), it is not likely that both LSOs contribute a maximum number of synapses to a given patch of neuropil. The ipsilateral LSO also sends inhibitory projections to pars lateralis of the central nucleus, where it can account for up to 26% of the terminals with pleomorphic vesicles (Oliver et al., 1995). Additional inhibitory input to the dorsolateral central nucleus arises in the periolivary nuclei as well as in the nuclei of the lateral lemniscus (e.g., Whitley and Henkel, 1984; Saint Marie and Baker, 1990; Bajo et al., 1999). In the gerbil, this part of the central nucleus appears to be a major target of the dorsal nuclei of the lateral lemniscus (Cant and Benson, 2006).

#### Input from the cochlear nuclei

In addition to the dense terminal plexuses formed by the inputs from the superior olivary complex, the lateral part of the central nucleus also receives substantial inputs from both the ipsilateral and contralateral cochlear nuclei. In fact, the pars lateralis is the main, if not the only, target of the ipsilateral cochlear nucleus in both cats (Oliver, 1987) and gerbils (Nordeen et al., 1983; Moore and Kitzes, 1985; Cant and Benson, 2006, 2008). In the gerbil, the inputs arise from both the ventral and dorsal cochlear nuclei (Nordeen et al., 1983; Cant and Benson, 2006, 2008). In the cat, only the ventral cochlear nucleus appears to contribute substantially to the projection (Oliver, 1987). The difference could be related to the relatively compressed low frequency representation in the cat dorsal cochlear nucleus (Spirou et al., 1993), especially when compared to that of the gerbil (Hancock and Voigt, 2001).

Like the inputs from the superior olivary complex, the inputs from the cochlear nuclei are not distributed homogeneously throughout their terminal zone, and the patchy inputs from the ipsilateral and contralateral sides may not fully overlap (Cant and Benson, 2008). The inputs from the cochlear nuclei form their densest terminations in the same part of the central nucleus that receives the inputs from the superior olivary complex (based on comparing the plots in Moore and Kitzes [1985] with those in Cant and Benson [2008] with those in the present results). Although the projections from the ipsilateral dorsal and ventral cochlear nuclei arise from a relatively small number of neurons (e.g., Nordeen et al., 1983), they form a relatively dense terminal field in the pars lateralis (Cant and Benson, 2008). Oliver (1984, 1985, 1987, 2000) found that the ipsilateral anteroventral cochlear nucleus can account for up to 18% of the excitatory terminals in the pars lateralis, whereas the contralateral anteroventral cochlear nucleus can account for up to 13% and the contralateral DCN, for up to 11%. Presumably, there is an additional contribution from the posteroventral cochlear nucleus, which also projects to pars lateralis (Cant and Benson, 2007, 2008).

#### Directions for further study: synaptic organization within pars lateralis

A major goal of neuroanatomy is to discover how specific populations of neurons are interconnected. The more precisely specific cell types can be defined in terms of their synaptic organization and projection patterns, the more useful the anatomical data will be for interpreting the results of physiological studies. For this reason, it is important not only to understand the circuitry but also to understand how the components are organized within the three-dimensional space of the structure under consideration. In the cochlear nuclei, tremendous progress has been made in correlating structure and function (reviewed, e.g., by Romand and Avan, 1997), in large part because of the physical segregation within the nucleus of many of the main cell types. In the central nucleus of the IC, progress is being made in identifying anatomically distinct cell types (e.g., Ito and Oliver, 2012), but these cell types are less obviously segregated within the structure. However, in many instances, the terminal fields formed by axons projecting from various sources into the central nucleus do appear to be partially or completely segregated from each other in some parts of the central nucleus and to converge in various combinations in others. Thus, precise mapping of the terminal fields of the inputs from the different sources can serve to constrain hypotheses about the circuitry in each region. The inputs to the gerbil IC from the LSO and MSO appear to intersect with each other and with the inputs from the cochlear nucleus (Cant and Benson, 2008) in a complex way with the potential for segregation of some of the inputs and overlap of others within the confines of this one subdivision. The pars lateralis is the part of the central nucleus in the cat that contains neurons sensitive to interaural delays (Semple and Aitkin, 1979), and the connections (as discussed above) suggest that the same would be true for the gerbil. Thus, the convergence in pars lateralis from most of the main sources of ascending excitatory input to the IC combined with a non-homogeneous distribution of the terminations suggests the potential for a number of different types of processing units within this one subdivision devoted to binaural integration. The pars lateralis gives rise to projections to at least two different regions within the ventral division of the medial geniculate nucleus (Cant and Benson, 2007), and it is quite possible that the projections arise from different cell populations with different complements of synaptic inputs.

A particularly important concept for guiding studies of the organization of the IC at the level of individual circuits is the concept of the “synaptic domain,” first articulated by Oliver and Huerta (1992). The central idea is that embedded within the frequency band laminae characteristic of the central nucleus is some number of functional modules, potentially definable on the basis of unique sources and arrangements of inputs and outputs. This is an attractive idea and evidence for it has been discussed in some detail by Oliver and colleagues (e.g., Oliver, 2000, 2005; Loftus et al., 2010). Given the wealth of possibilities for interactions among ascending sources within the pars lateralis, it seems like a particularly interesting part of the IC for continuing studies based on the hypothesis of synaptic domains.

#### Conflict of interest statement

The author declares that the research was conducted in the absence of any commercial or financial relationships that could be construed as a potential conflict of interest.
